# Impact of a home-based social welfare program on care for palliative patients in the Basque Country (SAIATU Program)

**DOI:** 10.1186/1472-684X-12-3

**Published:** 2013-01-30

**Authors:** Emilio Herrera Molina, Roberto Nuño-Solinis, Gorka Espiau Idioaga, Silvia Librada Flores, Naomi Hasson, Juan F Orueta Medía

**Affiliations:** 1 Enterprising Solutions for Health, SL. Galia Puerto. Carretera de la Esclusa, 11, CP.41014, Seville, Spain; 2 O+berri, Basque Institute for Healthcare Innovation, Basque Country, Spain; 3 Association for the Promotion of Innovation DENOKINN, Basque Country, Spain; 4 SAIATU Program Coordinator, Guipúzcoa, Spain

**Keywords:** Palliative care, Terminal care, End of life, Social support, Voluntary, Social needs, Cost effectiveness, Efficiency

## Abstract

**Background:**

SAIATU is a program of specially trained in-home social assistance and companionship which, since February 2011, has provided support to end-of-life patients, enabling the delivery of better clinical care by healthcare professionals in Osakidetza (Basque Health Service), in Guipúzcoa (Autonomous Community of the Basque Country).

In January 2012, a retrospective observational study was carried out, with the aim of describing the characteristics of the service and determining if the new social service and the associated socio-health co-ordination had produced any effect on the use of healthcare resources by end-of-life patients.

The results of a comparison of a cohort of cases and controls demonstrated evidence that the program could reduce the use of hospital resources and promote the continuation of living at home, increasing the home-based activity of primary care professionals.

The objective of this study is to analyse whether a program of social intervention in palliative care (SAIATU) results in a reduction in the consumption of healthcare resources and cost by end-of-life patients and promotes a shift towards a more community-based model of care.

**Method/design:**

Comparative prospective cohort study, with randomised selection of patients, which will systematically measure patient characteristics and their consumption of resources in the last 30 days of life, with and without the intervention of a social support team trained to provide in-home end-of-life care.

For a sample of approximately 150 patients, data regarding the consumption of public healthcare resources, SAIATU activity, home hospitalisation teams, and palliative care will be recorded. Such data will also include information dealing with the socio-demographic and clinical characteristics of the patients and attending carers, as well as particular characteristics of patient outcomes (Karnofsky Index), and of the outcomes of palliative care received (Palliative Outcome Scale).

Ethical approval for the study was given by the Clinical Research Ethics Committee of Euskadi (CREC-C) on 10 Dec 2012.

**Discussion:**

The results of this prospective study will assist in verifying or disproving the hypothesis that the in-home social care offered by SAIATU improves the efficiency of healthcare resource usage by these patients (quality of life, symptom control).

This project represents a dramatic advance with respect to other studies conducted to date, and demonstrates how, through the provision of personnel trained to provide social care for patients in the advanced stages of illness, and through strengthening the co-ordination of such social services with existing healthcare system resources, the resulting holistic structure obtains cost savings within the health system and improves the efficiency of the system as a whole.

## Background

In the developed world, some 10,000 people per million population die every year. Some 70% of the total population lives through a period of terminal illness lasting several months before their eventual death, whether it be a type of cancer (2,500 cases per million population) or the final stage of deterioration of non-oncological illnesses (approximately 4,500 cases per million population) [1]. It has been accepted internationally that the largest proportion of healthcare costs incurred by a citizen are generated in the final months of life. We are therefore discussing the largest source of costs to the healthcare system, an issue to which insufficient attention has been paid. In these cases, both the symptoms themselves and the complexity of accompanying circumstances cause a high degree of suffering in the patient and a social and family crisis in his immediate environment, as well as incurring the largest share of healthcare expenditure in the life of each respective patient.

Palliative Care (PC) [2] has been scientifically demonstrated as a truly effective tool in both welfare and organisational terms, complementing appropriate medication and medical care with psychological, social and spiritual support for patients and their careers.

In addition, the final period of illness is accompanied in nearly all cases by a more or less prolonged period of functional deterioration, leading inexorably to the development of a state of dependence on the part of the terminally ill patient, often accompanied by tremendous socio-familial complexity. Thus, the enormous diversity of psycho-social factors that surround every case can generate a wide range of needs, of greater or lesser severity, which need to be attended to routinely, and which, conversely, do not fall within the competencies provided by the health system itself. In fact, such needs are better understood within the social sphere and often include, among others:

- *Need for attention to patient dependency*: assistance with performing the basic and instrumental activities of daily living; reducing as far as possible the loss of sensory capabilities, and facilitating measures which can compensate for such deterioration; training in habits that improve personal autonomy; early warning of loss of autonomy; measures for the safety and protection of the patient; and adaptation of the environment.

- *Needs of carers and the patient’s social support network*: information about available support services; training and capacity-building for professional and/or family carers; development of communication skills to facilitate dialogue with the patient; family rest and respite; reconciliation of care with the professional life of the carer; psychosocial support to prevent burnout; and the exchange of experiences with other carers.

- *Protection of the patient’s social role:* decision-making autonomy and the communication of the final will; companionship; spiritual expression; leisure and entertainment; privacy or intimacy; interpersonal and social relationships.

While the health system is aimed at ensuring comprehensive care for patients and families, addressing those aspects related to healthcare, and encouraging valid solutions to social problems, reality demonstrates that this is often insufficient. When healthcare teams conduct in-home visits, a solid care structure is required, capable of performing all necessary tasks. Indeed, the problem is two-fold: increasingly, such structures are dwindling as a result of the crisis in informal caregivers, while the assistance required, due to patient complexity, calls for an increasing degree of skill.

In effect, changes to the socio-demographic structure, the ageing population, and the increasing incidence of chronic illness, have been accompanied by the weakening of traditional social support networks, diminishing the available number of informal caregivers who, historically, would have offered in-home care as a matter of course [3].

The development of in-home care from the social sector and its expected growth since the entry into force of the Law for the Promotion of Autonomy and Care for People in a Dependent Situation [4] are not yet sufficient to meet current assistance needs. Furthermore, in the absence of clearly defined alternatives, they will evidently be inadequate to provide family support and patient care for increasingly complex cases in the future, especially if improvements to training procedures are not implemented.

### Effectiveness and efficiency of palliative care at a global level

The incorporation of Support Teams for palliative patients into traditional models of patient care (primary attention, specialist care, emergency, residential centres) offers effectiveness in outcomes such as improving control of symptoms [5], reduction of health-care costs [6], appropriate process management, improvements in quality of life outcomes, and patient and family satisfaction [7,8].

At the hospital level, Palliative Care (PC) support teams act with the advice and support of clinical professionals to resolve the specific and complex problems of terminal patients and ensure co-ordination between levels of care. The incorporation of these specialised teams has been demonstrated to effectively reduce the length of hospital stays [9].

This is one of the most commonly used indicators to measure the cost effectiveness of Palliative Care teams. The reduction of average stay length in a hospital patient is directly correlated with a decrease in both the total and indirect costs of hospital care, which is often unnecessarily prolonged [10]. One study conducted in the United States demonstrated a reduction in costs of US$1.8 million per year after the introduction of PC teams in the hospital [9]. Another study, carried out in Spain, found a reduction in the average length of hospital stay, from 25.5 days to 19.9 days, which coincides with the averages of other studies [11]. This study was conducted over two different time periods, one in 1992 under a traditional care regime and one in 2001 with the presence of functional PC teams. Costs were reduced from 5,068 € per patient per year in 1992 under the traditional hospital model to 1,963 € per patient per year in 2001 [12].

The results of other studies conclude that a reduction of 40-70% in hospital costs could be achieved through the provision of specialised Palliative Care support teams [13]. In addition to providing adequate symptom control, the interventions of PC support teams reduced the number of medical tests and interventions, as well as offering support to families, occasionally allowing them to leave the hospital with the security of relying on an onsite support team [14].

Similar results have been encountered in hospital Palliative Care Units (PCUs), where the costs associated with patient care are lower than those in acute care hospitals. In Smith’s 2006 study [15], a cost reduction of 57% was achieved under the Palliative Care mode. Similarly, in a comparative study between PCUs and hospital units, the average cost per patient in the PCU was calculated at US$700 per day, compared with a cost of US $2,500 per day in Intensive Care units [16]. Significant reductions in hospital admissions have also been found in comparative studies in other countries [17]. In addition, as the PCUs are used in the most complex cases, the consistency and effectiveness demonstrated in the follow-up of patients in acute crisis has facilitated a reduction in hospital admissions, visits to emergency departments and intensive care units [17]. Early identification of patients in terminal stages and their transfer to specialised units allows for the appropriate planning of care and ensures its continuity, leading to effective control of symptoms, reduction of nonspecific treatments, and improvements in quality of life for both patients and their families

In the home environment, the teams offer support and advice to primary care professionals through consultation, direct assistance with the evaluation of patients and families, and the design of appropriate therapeutic intervention strategies.

This support ensures co-ordination between levels of care and improves the portfolio of primary care services [18] with regard to their responsiveness to the complexities of terminal patients. For its part, home-based palliative care has demonstrated effectiveness in:

- Reducing the number of hospital admissions, visits to emergency departments and other specialties, and hospital stays (reduction of 8 days on average) [19], which translates to a decrease in healthcare costs [20,21]. In addition, this type of care allows for a reduction in unnecessary visits to primary care providers and in the length of stays in residential centres.

- The highest rate of deaths at home, which translates to an improvement in the satisfaction and quality of life of both patient and family. In addition to generating emotional and psychological advantages and permitting better control of symptoms, death at home translates to a reduction in hospital costs, which can result in a saving of some 4,000 € for hospital stays per patient [22]. Today, the majority of deaths occur in hospital, which could entail greater economic costs (around 58% currently, and rising to 65% by the year 2030). This is despite evidence showing that between 56% and 65% of patients prefer to die at home [23].

Thus, it is evident that Palliative Care experiences are proving successful in terms of quality, effectiveness, efficiency and cost savings. Indeed, it would be reasonable to anticipate that, based on current socio-demographic trends regarding the prevalence of chronic illnesses and the lack of family caregivers at home, social healthcare development models should begin to promote appropriate social welfare support of community networks in order to make them genuinely useful to healthcare.

At first glance, this solution may appear unfeasible, given that the increased cost of providing special training for home-based assistants would be nearly impossible to contend with in the current environment of socio-economic crisis and budgetary cuts. Nevertheless, hypotheses that defend the efficiency of social healthcare could make it clear that, through investment in better social care, the health system could encounter part of the solution for better healthcare, and, through improved efficiency, reduce overall healthcare costs over time.

Despite the fact that the majority of patients within the Basque Country (60%) die in hospitals, hospitalisation does not always offer better quality of life. Furthermore, the practice of hospitalisation leads to the saturation of emergency services and intensive care units [19,24]. As has been demonstrated in other studies consulted in the literature, the majority of people prefer not to have to die in a hospital, which is cold, routine, impersonal, and high-cost. Rather, people have a strong preference for dying at home, which suggests that patient care should be focused there. This, in turn, would generate a societal demand for staff to support terminal stage patients at home.

### SAIATU program

The SAIATU in-home care program is a social innovation project launched in February 2011 in Guipúzcoa, with the aim of providing a set of in-home social support services to complement clinical palliative care, in order to improve comprehensive care for people with advanced and terminal illness and their families. This has entailed widening the scope of the classic model of primary care in palliative patients, expanding the traditional model to a cross-cutting action framework.

Currently, the program provides care in complex social situations, or in cases requiring attendance by clinical teams to provide appropriate symptom control, which requires the assistance of a social support network to facilitate the interventions of Osakidetza palliative care teams.

This new care model for Palliative Care patients forms part of an innovative approach, which aims to co-ordinate social services and healthcare in the field of palliative care. This approach is currently thriving in other health systems internationally, including the Canadian and British health systems, and seeks to provide both the best possible comprehensive care and efficiency in the provision of complementary health and social services [25].

SAIATU has been the first such experience in Spain, and the first internationally which combines the quantification, analysis and impact assessment of the reduction of healthcare resource usage by end-of-life patients, based on a pilot study of in-home social care for palliative care patients in the Basque Country.

The evaluation of the program, conducted in January 2012, has attempted to compare the difference in the intensity of health care provided to end-of-life patients in traditional services and in specialised Palliative Care services, but, for the first time, adding to the second group the effect of a social service trained in Palliative Care.

On the one hand, the pilot experience has been of enormous utility in properly channelling the program’s contribution to the real needs of the patients and their families, clarifying what should be the vision and mission of the program, and determining that SAIATU should position itself as a Specialised Social Program, in close co-ordination with the current health system (primary care, specialised care, and home hospitalisation).

On the other hand, the results of the pilot experience have yielded data suggesting that the SAIATU program:

- Reduces the consumption of health care resources on the part of program users.

- Facilitates staying at home for the patient, in compliance with patients’ preference for dying at home.

- Increases the number of home-based activities developed by Primary Care.

- Has yielded satisfactory outcomes for the families of patients questioned in the course of the study.

These results are highly striking and, if confirmed, would be of tremendous importance for improving the efficiency of the health system, and for the development of models for complementary action between the social and health sectors. However, with the current work, the results should be treated as the results of a descriptive and comparative study, retrospective in nature, and thus the scientific strength of the results is highly relative.

For this reason, a prospective study with greater sample size, enabling the validation with sufficient strength and validity of the results obtained in this work, is considered of great interest to society in general and the Basque country in particular. Such a study would be one of the first to provide clear evidence of the efficiency gains offered by complementary and co-ordinated action in the social and health sectors and, without doubt, the first worldwide in the field of palliative care.

### General objective

To analyse whether a program of social intervention in palliative care (SAIATU) results in a decrease in the consumption of healthcare resources and cost by end-of-life patients, and produces a shift towards a more community-based model of care.

### Specific objectives

- Describe the average profile of healthcare resource consumption of a retrospective cohort (patients who died from malignant neoplasm) in the last 30 days of life by diagnosis and age group.

- Quantify and compare the costs of the use of healthcare resources in the cohorts of patients with and without SAIATU intervention.

- Compare quality of life in the cohorts of patients with and without SAIATU intervention.

## Methods/design

### Hypothesis

The intervention of SAIATU (a resource for the social support of end-of-life patients) improves efficiency in the use of healthcare resources in end-of-life patients, decreasing the consumption of hospital resources and increasing the home-based activities conducted by Primary Care in the last 30 days of life.

### Location and date of study

The study will be conducted in the provinces of Guipúzcoa.

Period of study: September 2012 - October 2013.

### Study design

The study was designed in two phases.

#### Phase 1: RETROSPECTIVE study to register a control cohort of patients who died from malignant neoplasm

The objective of this study is to determine the baseline risk of the principal variable, *consumption of resources* in the population of patients who die from malignant neoplasm.

Thus, the study characterises, by primary diagnosis (criteria and rules established by the International Statistical Classification of Diseases 10 [ICD-10] [26]) and age, the behaviour of different malignant neoplasms, with regard to resource consumption: number of visits to or consultations with Primary Care, number of external consultations, number of visits to hospital emergency departments, number of hospital admissions, average length of stay, and days in home hospitalisation.

Time of study: time series of 4 years, to be determined based on Osakidetza records and the mortality register.

Based on the results of this study and on the hypothesis of the decrease in, and improved efficiency of, resource consumption through the intervention of support programs, formulated as a result of the retrospective observational study carried out, the sample size of cohorts and subgroups will be defined for Phase 2: PROSPECTIVE cohort study.

For this analysis, the error will be established at α = 5% with a statistical significance of 80% (error β = 20%). The sample size of each subgroup *n* will be the maximum of the sample sizes obtained for the comparison of means or proportions of the main variables in each subgroup.

For the comparison of two means:

(1) $$n\;=\;\left(2\left[\left(\mathrm{Z\alpha}/2+\mathrm{Z\beta}\right)\right]\wedge 2*\text{S}\wedge 2\right)/\text{d}\wedge 2$$

where:

(2) $$\begin{array}{l} \mathrm{Z\alpha}/2\;=\;1.960\\ \mathrm{Z\beta}\;=\;0.842 \end{array}$$

S^2^ = variance in the quantitative variable of the control group

d = Minimum value of the difference to be detected (quantitative values)

For the comparison of two proportions:

(3) $$n\;=\;2p*q\wedge 2\left(\mathrm{Z\alpha}/2+\mathrm{Z\beta}\right)\wedge 2/\left(\mathrm{pA}-\mathrm{pB}\right)\wedge 2$$

where:

(4) $$\begin{array}{l} \mathrm{Z\alpha}/2=1.960\\ \mathrm{Z\beta}=0.842 \end{array}$$

p = Mean of the two proportions pA y pB

The parameters will be analysed with the statistical software SPSS 15.0 for Windows.

A descriptive study will be conducted on the consumption of healthcare resources by diagnosis and age group in the last 30 days of life. The descriptive study will include measures of central tendency (mean), confidence intervals at 95% for the population mean, and contingency tables (frequencies) for each recorded variable.

#### Phase 2. Study of PROSPECTIVE cohorts, assigned by randomised blocks

The study will be initiated with two cohorts:

1. ***Exposed cohort*:** comprising patients who will be attended by SAIATU, without prejudice to the care received from the public health system.

2. ***Control cohort*:** comprising patients who will be cared for exclusively through the public health system.

The evolution of the disease over the course of the study will determine the different healthcare mechanisms (Primary Care, Specialised Care (SC), Home Hospitalisation (HH)) that will intervene in the care of each patient, so that, at the end of the study, the two initial cohorts will be divided into 4 subgroups:

1) *Exposed cohort*

1.1. Patients attended by Primary Care + SC + SAIATU

2.2. Patients attended by Primary Care + SC + HH + SAIATU

**Figure 1:**
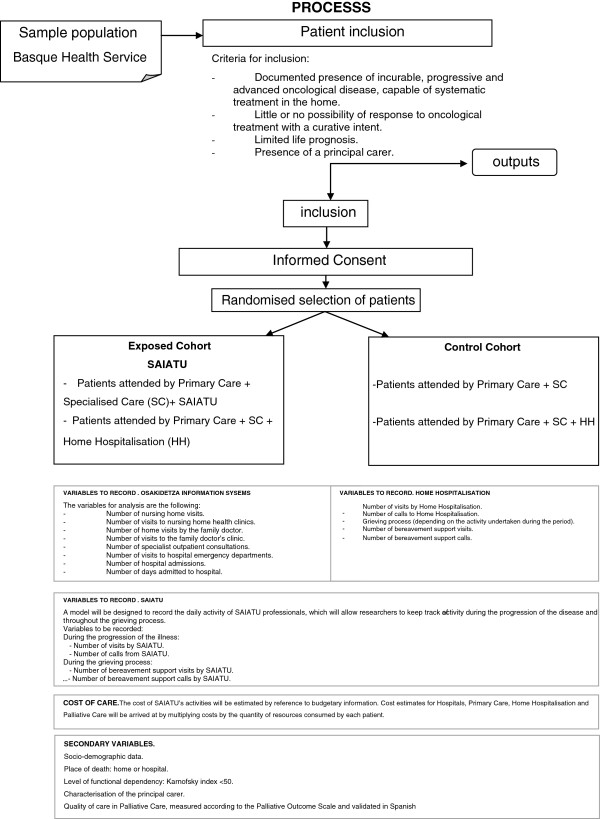
**Flow chart.** Study of prospective cohorts, assigned by randomised blocks.

2) *Control cohort*

a. Patients attended by Primary Care + SC

b. Patients attended by Primary Care + SC + HH (Figure 1)

### Population for Study

This study will comprise oncology patients from the health districts of Guipúzcoa, attended by the Basque Health Service.

Assuming that 2,500 people per million population die each year from malignant neoplasm, and taking into account that the population of Guipúzcoa is 709,607 inhabitants, it is estimated that the eligible population for this study is 1,774 deaths from malignant neoplasm per year. For a period of 10 months of prospective study, this corresponds to a population of 1,478 deaths from malignant neoplasm in these provinces.

Although the final number of patients for inclusion will be calculated during the design of the prospective study itself, after the results of Phase 1, it is estimated that the resulting sample size could allow some 150 patients to be studied. This sample, which would correspond to 10% of the total population of deaths from cancer in this period, would strengthen the study’s feasibility with respect to its representativeness of the total population in both provinces.

### Criteria for inclusion and exclusion from the study sample population

Criteria for inclusion:

- Documented presence of incurable, progressive and advanced oncological disease, capable of systematic treatment in the home.

- Little or no possibility of response to oncological treatment with a curative intent.

- Limited life prognosis.

- Presence of a principal carer.

Criteria for exclusion:

- Death or disability of the family carer.

- Withdrawal from the Basque public health system

- Absence of compliance with informed consent.

### Variables and sources of information

#### Main variable: Consumption of resources

##### Primary and specialised care

Records of patient activity will be obtained from Osakidetza’s information systems.

The variables for analysis are the following:

- Number of nursing home visits.

- Number of visits to nursing home health clinics.

- Number of home visits by the family doctor.

- Number of visits to the family doctor’s clinic.

- Number of specialist outpatient consultations.

- Number of visits to hospital emergency departments.

- Number of hospital admissions.

- Number of days admitted to hospital.

##### Home hospitalisation

A formula will be designed to record the daily activity of Home Hospitalisation professionals during the period of care.

Variables to record:

- Number of visits by Home Hospitalisation.

- Number of calls to Home Hospitalisation.

- Grieving process (depending on the activity undertaken during the period).

◦ Number of bereavement support visits.

◦ Number of bereavement support calls.

### SAIATU

A model will be designed to record the daily activity of SAIATU professionals, which will allow researchers to keep track of activity during the progression of the disease and throughout the grieving process.

Variables to be recorded:

- During the progression of the illness:

◦ Number of visits by SAIATU.

◦ Number of calls from SAIATU.

- During the grieving process:

◦ Number of bereavement support visits by SAIATU.

◦ Number of bereavement support calls by SAIATU.

### Costs of care

The cost of SAIATU’s activities will be estimated by reference to budgetary information. Cost estimates for Hospitals, Primary Care, Home Hospitalisation and Palliative Care will be arrived at by multiplying costs by the quantity of resources consumed by each patient.

Where budgetary information about the activities of Hospitals, Primary Care and Home Hospitalisation is available, a calculation will be made multiplying costs by the quantity of resources consumed by each patient.

Secondary variables:

- Socio-demographic data.

- Place of death: home or hospital.

- Level of functional dependency: Karnofsky index <50.

- Characterisation of the principal carer.

- Quality of care in Palliative Care, measured according to the Palliative Outcome Scale and validated in Spanish [27].

### Analysis of variables

The parameters will be analysed using the statistical software SPSS 15.0 for Windows.

A descriptive study will be conducted on the *consumption of healthcare resources* by subgroup in the last 30 days of life. The descriptive study will include measures of central tendency, confidence intervals at 95% for the population mean, and contingency tables (frequencies) for each of the recorded variables.

Each subgroup will be analysed by age, sex, and main diagnosis.

For the comparison of proportions, Pearson’s Χ^2^ test, or, when appropriate, Fisher’s exact test will be used to calculate relative risk (RR), absolute risk reduction (ARR) and the number needed to treat (NNT) with 95% confidence interval.

The Student T-test will be applied to calculate the equality of means for independent samples in the case of continuous variables, upon confirmation of the conditions of normality and homoscedasticity.

The quantification of costs incurred by the SAIATU program will be calculated on the basis of the activities performed by the program’s professionals, measured in hours of care. Based on the actual time spent on patients, the total cost will be distributed between each of the activities.

Calculations of unit costs per activity, taking into account the number of times each activity is performed for the period under analysis, will finally be performed.

### Ethical considerations

Ethical approval for the study was given by the Clinical Research Ethics Committee of Euskadi (CRE-C) on 10 Dec 2012.

## Discussion

SAIATU could become a benchmark for an innovative model of home-based palliative care, focusing on the complementary aspects of healthcare; namely, social welfare and companionship. It is the first program to define a specific portfolio of services directed at the social welfare and support of end-of-life patients. This will allow the creation of new professional profiles to carry out this type of work, as well as clarifying which capabilities should be fostered in the training of in-home support staff, so that in the future they will be able to care for patients with advanced disease and high levels of dependency (a basic tool to cope with the socio-demographic changes we are currently undergoing).

The current project could be a graphic demonstration of an important solution to improved efficiency in the health system, through investment in resources outside the health system; in this case, in the social sector. Should the current hypothesis be confirmed, the creation of a reserve of appropriately trained home care professionals would lead to a more community-based model of healthcare, resulting in a more economical expenditure of the total resources used in the integrated care process.

If there is evidence of a difference between the cohorts under study, it would be possible to estimate, based on the incidence of the various diseases, the cost savings that could be achieved through a program of professional training and communal resource procurement if the project was extended the project to the entire Basque territory.

Taken as a whole, the results will allow for an analysis of how the development of skilled social resources and socio-health co-ordination, when applied to groups of end-of-life patients, strengthens the overall efficiency of both systems: healthcare and social welfare.

It is expected that the study will stratify oncological patients by iso-resource consumption, according to the average profile of end-of-life behaviour.

It will be possible to assess the consumption of healthcare resources at different levels of care, with and without the intervention of the SAIATU program, quantifying the similarities and differences both in terms of activities undertaken and in economic terms. Furthermore, it will be possible to examine if there are statistically significant differences in a study of sufficient methodological strength and robustness.

Indirectly, the study will generate:

- Organisational and operational protocols for programs such as SAIATU in the future.

- Work systems which utilise liaison committees for assessment and co-ordination between different levels of care and across sectors.

- Protocols for the primary assessment of the needs of end-of-life patients, which take into account both clinical and social aspects, supplementing the assessment of the illness itself with an account of patient’s degree of suffering, their level of dependence, and their social support network, thus allowing for better allocation of resources.

- Criteria for referrals between professionals of different levels, according to the needs of patients and their families.

## Abbreviations

PC: Palliative care; PCU: Palliative care unit; HH: Home hospitalisation.

## Competing interests

The authors declare that they have no competing interests.

## Authors’ contributions

The idea for this study was initially conceived by RN, EH, and GE. EH, JO and SL designed the study’s methodology and contributed to the analysis of a previous retrospective study on the SAIATU experience. NH coordinated the interventions, as well as the inclusion of patients and variables in the study. All authors have given final approval of the version submitted.

## Pre-publication history

The pre-publication history for this paper can be accessed here:

http://www.biomedcentral.com/1472-684X/12/3/prepub
